# Surfactin Facilitates Horizontal Gene Transfer in *Bacillus subtilis*

**DOI:** 10.3389/fmicb.2021.657407

**Published:** 2021-05-14

**Authors:** Tjaša Danevčič, Anna Dragoš, Mihael Spacapan, Polonca Stefanic, Iztok Dogsa, Ines Mandic-Mulec

**Affiliations:** ^1^Chair of Microbiology, Department of Food Science and Technology, Biotechnical Faculty, University of Ljubljana, Ljubljana, Slovenia; ^2^Bacterial Interactions and Evolution Group, Department of Biotechnology and Biomedicine, Technical University of Denmark, Kongens Lyngby, Denmark

**Keywords:** quorum sensing, competence, surfactin, DNA exchange, extracellular DNA, cell lysis, horizontal gene transfer

## Abstract

Genetic competence for the uptake and integration of extracellular DNA is a key process in horizontal gene transfer (HGT), one of the most powerful forces driving the evolution of bacteria. In several species, development of genetic competence is coupled with cell lysis. Using *Bacillus subtilis* as a model bacterium, we studied the role of surfactin, a powerful biosurfactant and antimicrobial lipopeptide, in genetic transformation. We showed that surfactin itself promotes cell lysis and DNA release, thereby promoting HGT. These results, therefore, provide evidence for a fundamental mechanism involved in HGT and significantly increase our understanding of the spreading of antibiotic resistance genes and diversification of microbial communities in the environment.

## Introduction

Surfactin is an important microbial surfactant with interesting biological activities. It is important for social spreading on solid surfaces (Kearns and Losick, 2003; Kinsinger et al., 2003; Grau et al., 2015; van Gestel et al., 2015), rhizosphere colonization (Bais et al., 2004; Aleti et al., 2016), biocontrol of a plant pathogen (Bais et al., 2004), or potentially even human viruses (Yuan et al., 2018). Its synthesis is regulated by a process called quorum sensing (QS) (Roggiani and Dubnau, 1993). In the Gram-positive bacterium *Bacillus subtilis*, the major QS system ComQXPA activates the transcription of hundreds of genes, including the *srfA* operon and competence genes (Weinrauch et al., 1990; Roggiani and Dubnau, 1993; Magnuson et al., 1994). The *srfA* operon encodes enzymes for the synthesis of surfactin and also contributes to the development of genetic competence (D’Souza et al., 1994; Hamoen et al., 2003; Comella and Grossman, 2005) through a small out-of-frame gene, *comS*, that is embedded in the second gene (*srfAB*) of the operon (Nakano et al., 1991; D’Souza et al., 1994; Hamoen et al., 2003; Comella and Grossman, 2005). ComS stops proteolytic degradation of ComK (Turgay et al., 1998) that then increases in concentration and consequently activates the transcription of late competence genes that encode processes responsible for the DNA uptake and integration (Dubnau, 1991). This state has been referred to as the K-state (Berka et al., 2002). Therefore, surfactin is linked to competence development because phosphorylated ComA (ComA-P) de-represses the *srfA* operon, which ultimately stabilizes ComK and the K-state, thereby increasing the transformation frequency of the population (Miras and Dubnau, 2016). Competence and transformation of *Bacillus subtilis* is a result of a complex regulatory network that, through stochastic cell differentiation, occurs only in 10–20% of the cells within a population and is transient (approximately 2 h) in nature (reviewed by Maier, 2020). It has been shown previously that in *B. subtilis* PS-216, competence is developed in the late exponential growth phase (Miras and Dubnau, 2016), when surfactin production is high (Oslizlo et al., 2014; Dogsa et al., 2021). Both ComA and the *srfA* de-repression, therefore, have significant effects on HGT, which shapes microbial evolution and ecology. Transformation also requires extracellular DNA (eDNA), which is spontaneously released by *B. subtilis* during growth (Zafra et al., 2012) through a mechanism that is not yet fully understood.

The aim of this study was to investigate the role of surfactin in eDNA release and genetic transformation. This is of fundamental importance as transformation mediates horizontal gene transfer and potentially contributes to dissemination of antibiotic resistance and diversification of microbial communities.

## Materials and Methods

### Bacterial Strains and Growth Conditions

Bacterial strains used in this study are listed in Table 1. Overnight cultures were incubated in liquid lysogeny broth (LB) with the appropriate antibiotics at 37°C and 200 rpm. The concentrations of antibiotics were as follows: chloramphenicol (Cat) 10 μg ml^–1^, spectinomycin (Spec) 100 μg ml^–1^, kanamycin (Kn) 50 μg ml^–1^, erythromycin (Ery) 0.5 μg ml^–1^, lincomycin (Lin) 12.5 μg ml^–1^, and ampicillin (Amp) 100 μg ml^–1^. Growth of *B. subtilis* strains was assessed by measuring optical density at 650 nm (OD_650_) following inoculation of fresh CM medium (Albano et al., 1987) with an overnight culture (1%, V/V) and incubation at 37°C and 200 rpm.

**TABLE 1:** Strains used in this study.

**Strain name**	**Descriptive**	**Background**	**Genome description**	**References**
***Bacillus subtilis* strains**				
PS-216	wt		Undomesticated strain	Stefanic and Mandic-Mulec, 2009
BM1060	wt^cat^	PS-216	*cotA:cat*	This work
BM1058	wt^spec^	PS-216	Δ*skfA:spec*	This work
BM1044	Δ*srfA*	PS-216	*srfA:*Tn*917* (mls)	This work
BM1062	Δ*srfA*^cat^	PS-216	*srfA:*Tn*917* (mls) *cotA:cat*	This work
BM1063	Δ*srfA*^spec^	PS-216	*srfA:*Tn*917* (mls) Δ*skfA:spec*	This work
BM1298	wt^lacZ^	PS-216	*amyE:*P*_43_-lacZ* (Kn)	This work
BM1299	Δ*srfA*^lacZ^	PS-216	*srfA:*Tn*917* (mls) *amyE:*P*_43_-lacZ* (Kn)	This work
BM1097		PS-216	*amyE:*P*_*hyperspank*_*-*mKate2* (Cm)	Stefanic et al., 2015
RL50		PY17	*trpC2 cotA:cat*	Donovan et al., 1987
EG165		PY79	Δ*skfA:spec*	Liu et al., 2010
OKB120		168	*pheA1 sfp srfA:Tn917* (mls)	Nakano et al., 1988
***Escherichia coli* strains**				
EM1070	Pkm3-p43-YFP	DH5α	*amyE:*P*_43_*-*yfp* (Spec, Amp)	Stefanic et al., 2015
EM1054	pBTK2	DH5α	pBKT2 *amyE:lacZ* (Kn, Amp)	Meijer et al., 1995
EM1055	pEM1055	DH5α	pBKT2 *amyE:*P*_43_-lacZ* (Kn, Amp)	This work

### Strain Construction

Mutant strains were constructed by transformation of specific markers into competent *B. subtilis* strains. Strains were grown in CM medium at 37°C and 200 rpm, and transformants were selected by antibiotic selections on LB agar plates with the appropriate antibiotics at 37°C. The Δ*cotA*, Δ*skfA*, and Δ*srfA* mutants were constructed by transforming appropriate *B. subtilis* strains with chromosomal DNA isolated from *B. subtilis* RL50 (Donovan et al., 1987), EG165 (Liu et al., 2010), or OKB120 (Nakano et al., 1988), respectively. The *amyE:*P*_43_*-*lacZ* mutants were constructed by transforming appropriate *B. subtilis* strains with plasmid DNA pEM1055.

To construct pEM1055 plasmid, carrying *amyE:*P*_43_*-*lacZ*, a constitutive promoter P*_43_* was PCR-amplified from plasmid DNA Pkm3-p43-yfp (Stefanic et al., 2015) using the primer pair p43-F1-*Eco*RI/p43-R1-*Bam*HI (Table 2). The PCR fragment was then digested with *Eco*RI and *Bam*HI and ligated into *Eco*RI and *Bam*HI sites of pBKT2 (Meijer et al., 1995). The constructed plasmid was then transformed into competent *E. coli* DH5α cells, and transformants were selected on LB agar plates containing 100 μg ml^–1^ Amp after overnight incubation at 37°C. Plasmids were isolated and screened by PCR using the same primer pair listed above to determine if the cells carried the *amyE:*P*_43_*-*lacZ* construct before transformation in *B. subtilis* strains.

**TABLE 2:** Oligonucleotides used in this study.

**Oligonucleotide name**	**Sequence 5′-3′**	**References**
p43-F1-*Eco*RI	CGCGAATTCTGATAGGTGGTATGTTTTCGCTTG	Stefanic et al., 2015
p43-R1-*Bam*HI	GCGGGATCCCCTATAATGGTACCGCTATCAC	This work

### Transformation Frequency Determination

Overnight cultures (1%, V/V) were inoculated into the fresh CM medium, and cells were grown at 37°C and 200 rpm for 6 h. After incubation, 1 ml of the culture was transferred to a glass tube and supplemented with genomic DNA of *B. subtilis* PS-216 *amyE:*P*_*hyperspank*_*-*mKate2* carrying chloramphenicol resistance (final saturating concentration 1.4 μg ml^–1^) (Stefanic et al., 2015). Tubes were incubated for 30 min at 37°C and 200 rpm. Fresh LB medium (0.5 ml) was then added, and samples were incubated for a further 60 min at 37°C and 200 rpm. Finally, 100 μl of sample dilutions (10^0^, 10^–1^, 10^–2^) were inoculated onto LB agar containing 10 μg ml^–1^ of Cat. In addition, a control experiment without DNA added was performed. Viable cell concentration was determined by standard CFU assay on LB agar plates without antibiotics. Transformation frequency was calculated as the ratio between the number of transformants and the viable cell number.

### Transformation Frequency Determination in Co-culture

Two *B. subtilis* strains carrying different antibiotic markers (*cat* or *spec*) integrated at different chromosomal loci in each strain were co-cultured. The co-culture approach enabled us to directly select for transformants with double resistance. Starting ratios of Cat^R^ strain and Spec^R^ strain (1:1) were prepared based on OD_650_ estimation of overnight cultures. Co-cultures were incubated in CM medium without or with surfactin (20 μg ml^–1^) and without or with DNase I (100 U) for 8 h at 37°C and 200 rpm, which allowed spontaneous integration of *cat* or *spec* antibiotic markers into Spec^R^ or Cat^R^ strains, respectively. After incubation, 100 μl of co-culture dilutions (10^0^, 10^–1^, 10^–2^) were plated onto LB agar plates containing two antibiotics, Cat (5 μg ml^–1^) and Spec (50 μg ml^–1^). Viable cell number in co-cultures was determined by standard CFU assay on LB agar without antibiotics. Transformation frequency in co-culture was calculated as a ratio of the number of transformants with both antibiotic markers and viable cell number.

### Influence of Surfactin on Growth and eDNA Release

Fresh CM medium was inoculated with an overnight culture (1%, V/V) of the Δ*srfA* mutant and incubated at 37°C and 200 rpm for 8 h. CM medium was supplemented with different surfactin concentrations (2.5, 5, 10, 15, and 20 μg ml^–1^). As controls, the wild-type strain and Δ*srfA* mutant were grown in the same conditions without surfactin addition. After incubation, cultures were centrifuged at 10,000 × *g* for 10 min, supernatants were filter sterilized, and eDNA concentration was determined. In addition, viable cell number was determined as described above.

### Time of Exposure to Surfactin

Fresh CM medium was inoculated with an overnight culture (1%, V/V) of the wt*^*lacZ*^* or Δ*srfA*^*lacZ*^ strain, and cultures were grown to an early stationary phase at 37°C and 200 rpm. Cultures were then supplemented with 20 μg ml^–1^ of surfactin and incubated for a further 3 h at room temperature without shaking. At different times of exposure to surfactin (0, 20, 40, 80, and 160 min), β-galactosidase activity and eDNA concentrations were measured in filter-sterilized conditioned medium of both strains, and viable cell number was determined as described above.

### β-Galactosidase Activity Measurement

Z-buffer (36 mM NaH_2_PO_4_, 67 mM NaHPO_4_, 0.1 mM MgCl_2_, 2 mM MgSO_4_) (25 μl) containing 5.6% (V/V) of β-mercaptoethanol and 10 μl toluene was added to the conditioned media of the wt*^*lacZ*^* and Δ*srfA*^*lacZ*^ strains in a microtiter plate, and the plate was incubated on ice for 30 min. The plate was then warmed to 30°C, then 50 μl of ortho-nitrophenyl-β-galactoside (ONPG) substrate was added, and the absorbance at 420 nm was measured during incubation for 15 min at 1-min intervals at 30°C using Multiscan Spectrum (Thermo Scientific). OD_650_ and ONPG degradation rate was used to calculate β-galactosidase activity.

### Extracellular DNA (eDNA) Concentration Determination

Extracellular DNA concentration in filter-sterilized conditioned media of the wild-type strain, Δ*srfA* strains following addition of different surfactin concentrations, and the wt*^*lacZ*^* and Δ*srfA*^*lacZ*^ strains and co-cultures was determined using QuantiFluor^®^ dsDNA system (Promega, United States) by measuring fluorescence intensity according to the manufacturer’s instructions. The excitation and emission wavelengths were set to 504 and 531 nm, respectively, and the gain was set to 100. A standard curve was prepared using the Lambda DNA standard provided with the kit. eDNA concentration was calculated by subtracting fluorescence intensity of an uninoculated growth medium (containing yeast extract) from fluorescence intensity of samples of conditioned media.

### Statistical Analysis

All data are presented as means and standard errors of biological replicates. Results were statistically evaluated using one-way ANOVA followed by Bonferroni’s *post hoc* comparisons tests using the *p* ≤ 0.05 level of significance.

## Results

### Genetic Transformation via DNA Exchange

**FIGURE 1:**
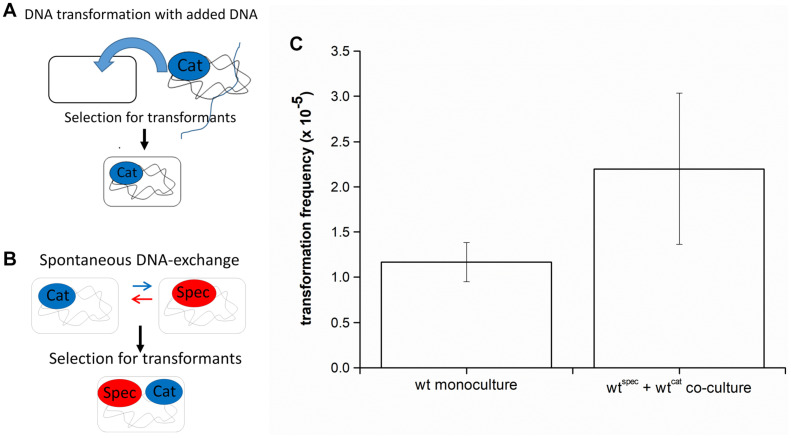
**(A)** A schematic representation of the transformation assay with added DNA. *Bacillus subtilis* strain was grown in monoculture in CM medium for 6 h at 37°C and 200 rpm and then genomic DNA carrying an antibiotic resistance marker (chloramphenicol-Cat^R^) was added to the medium. This enabled cells to receive DNA and provided an opportunity to develop resistance to antibiotic (Cat-Cat^R^). **(B)** A schematic representation of the DNA exchange assay. Two *B. subtilis* strains, each carrying a different antibiotic resistance marker (chloramphenicol-Cat^R^ or spectinomycin-Spec^R^) were grown in co-culture in CM medium for 8 h at 37°C and 200 rpm. This enabled spontaneous DNA exchange between the two strains and provided an opportunity to develop resistance to both antibiotics (Cat and Spec-Cat^R^Spec^R^). **(C)** Transformation frequency of *Bacillus subtilis* PS-216 wild-type strain in monoculture and in co-culture grown in CM medium. The values presented are means and standard errors (*n* = 3).

Genetic transformation is mostly studied in monocultures to which extracellular DNA with an antibiotic resistance locus is added, and transformants are selected on LB agar plates containing this antibiotic (Figure 1A). However, in a natural setting, DNA is usually released by cells that undergo lysis or actively release DNA (reviewed in Ibáñez de Aldecoa et al., 2017), which could be captured by cells in the vicinity (Steinmoen et al., 2002; Veening and Blokesch, 2017). This assumption was tested by mixing two *B. subtilis* PS-216 strains, which carry different antibiotic markers (chloramphenicol or spectinomycin resistance cassettes/cat or spec) integrated at different chromosomal loci, at 1:1 ratio. After the indicated incubation time, transformants were selected by plating the co-culture directly on LB agar plates with both antibiotics (Figure 1B). We refer to this process as DNA exchange because both single-antibiotic resistance strains have a comparable chance to acquire the resistance gene of the second antibiotic, thereby becoming double-antibiotic resistant. The results show that transformation frequency by DNA exchange is high [(2.2 ± 0.84) × 10^–5^]. If the standard transformation assay was carried out by adding the saturating concentration of DNA to PS-216 monoculture, the detected transformation frequency was (1.17 ± 0.22) × 10^–5^ (Figure 1C). Next, it was tested whether DNA exchange is mediated by transformation by adding DNase I (100 U) to the co-culture and again selecting transformants for Cat^R^Spec^R^double resistance. DNA exchange of PS-216 wild-type strains in co-culture was completely abolished in the presence of DNase I. The fact that we could not detect any transformants in the presence of DNase I indicates that DNA exchange is mediated by free DNA and not horizontal gene transfer vesicles.

### Surfactin Promotes DNA Release and Facilitates Intraspecies DNA Exchange

**FIGURE 2:**
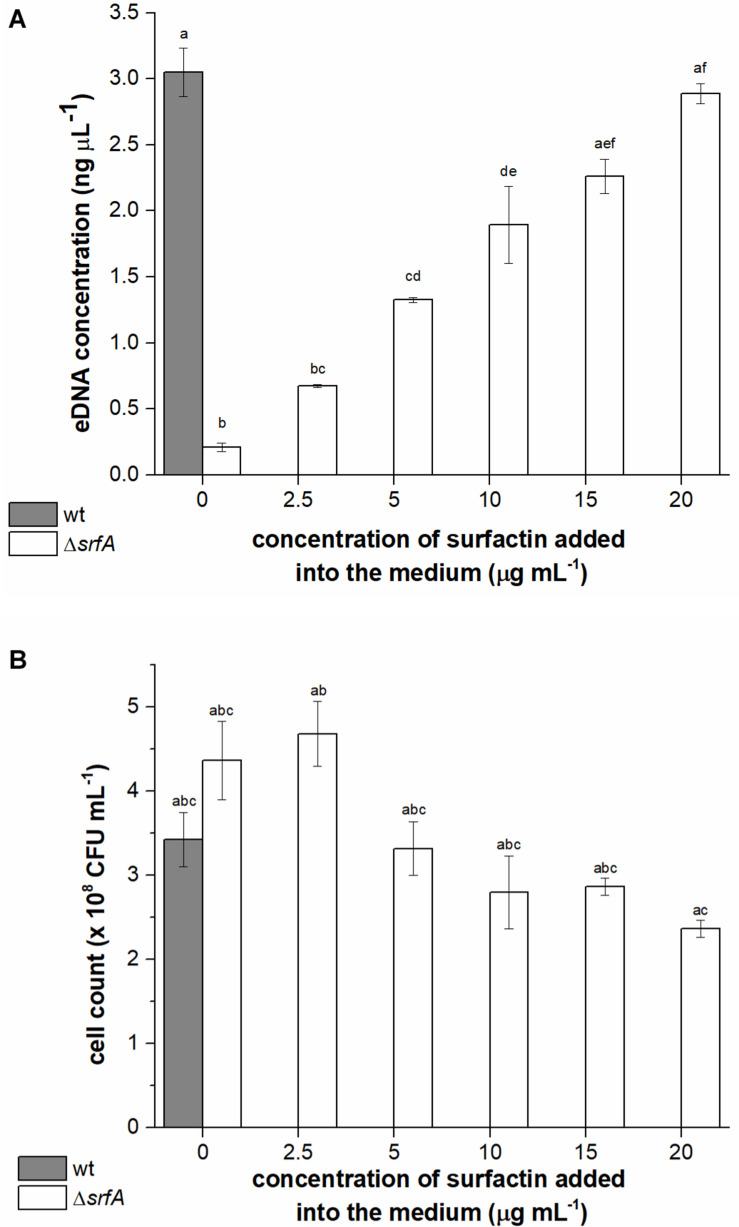
Extracellular DNA (eDNA) concentrations in the conditioned medium **(A)** and cell count **(B)** of *Bacillus subtilis* wild-type strain (PS-216) (gray column) and Δ*srfA* mutant (BM1044) (white columns) grown in CM medium for 8 h at 37°C and 200 rpm. Mutant Δ*srfA* was supplemented with different concentrations of surfactin (ranging from 2.5 to 20 μg ml^–1^). The values presented are means and standard errors (*n* = 3). Statistical significance was determined using one-way ANOVA followed by Bonferroni’s *post hoc* comparisons tests (*p* < 0.05) to do pairwise comparison between two strains (wt and Δ*srfA* mutant) and between treatments (different concentrations of surfactin added to the mutant). Different letters above the columns indicate a statistically significant difference between mean values across all columns. If treatments have the same letter, the difference between the mean values is not statistically significant.

It is already known that *B. subtilis* NCIB 3610 secretes high molecular weight DNA during growth (Zafra et al., 2012) and that *B. subtilis* strains with a deletion in the surfactin operon (*B. subtilis* NCIB 3610 Δ*srfAA*) are impaired in DNA release (Zafra et al., 2012). However, whether surfactin may have a direct or indirect role in this process has not been tested directly. To determine the relationship between surfactin concentration and DNA release, the Δ*srfA* mutant (BM1044) was incubated with different concentrations of surfactin (2.5-20 μg ml^–1^) and eDNA concentration was compared with that released by the wild-type strain. eDNA concentration in the conditioned medium of the Δ*srfA* mutant strain was approximately 15-fold lower than that in the conditioned medium of the wild-type strain (Figure 2A). We also observed that levels of eDNA released by the Δ*srfA* mutant could be restored by the addition of surfactin to the growth medium, in a concentration-dependent manner (Figure 2A). The maximal surfactin concentration (20 μg ml^–1^) recovered the phenotype of the surfactin mutant to the levels of the surfactin producing wild-type strain. This surfactin concentration was previously measured in the spent media of *B. subtilis* PS-216 during early stationary phase (Oslizlo et al., 2014). It is known that when the surfactin concentration is below or near the critical micelle concentration (10–25 μg ml^–1^) surfactin inserts into phospholipid layers inducing mild content leakage. On the other hand, at higher concentrations, surfactin attacks the phospholipid bilayer causing membrane solubilization (Deleu et al., 2013). The influence of surfactin concentration on final colony-forming units (CFU) was determined for a Δ*srfA* mutant where CFU count did not decrease significantly with increasing surfactin concentration (Figure 2B). These results, therefore, indicated that surfactin promotes eDNA release, either through active release that might limit cell growth (although this is not easily detectable) or through cell lysis.

**FIGURE 3:**
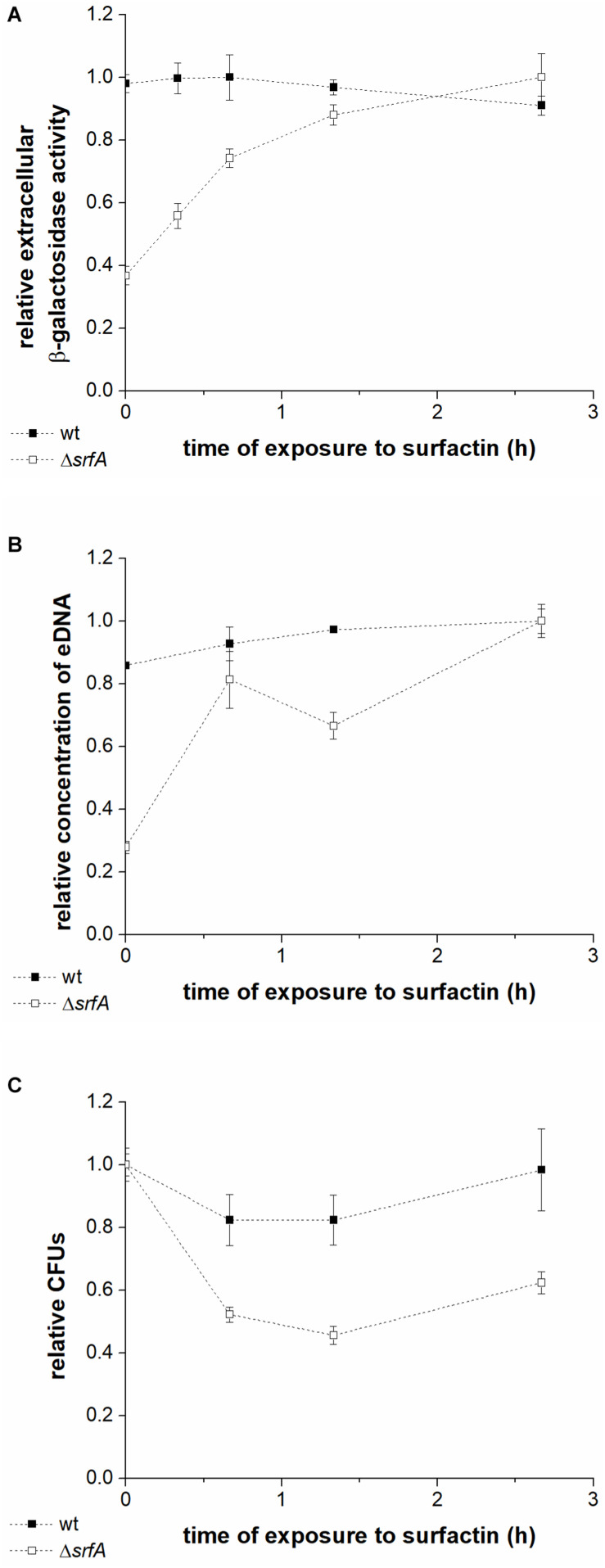
Extracellular β-galactosidase activity in the conditioned medium **(A)**, DNA released into the medium **(B)**, and colony-forming units **(C)** in the wt^lacZ^ (BM1298) (black squares) and Δ*srfA*^lacZ^ (BM1299) (white squares) strains after different lengths of exposure to surfactin. Both strains were grown until early stationary phase in CM medium at 37°C and 200 rpm and were then incubated with surfactin (20 μg ml^–1^) for 3 h at room temperature without shaking. Data were normalized with respect to the highest β-galactosidase activity **(A)**, eDNA concentration **(B)**, or CFU number **(C)** of all samples measured for a particular strain, and are presented in relative units. Values are presented as means and standard errors (*n* = 3).

To further investigate whether cell lysis is responsible for eDNA release, levels of extracellular β-galactosidase in the conditioned medium of wt^lacZ^ (BM1298) or Δ*srfA*^lacZ^ (BM1299) cultures were measured after incubation with surfactin (Figure 3A). β-galactosidase is localized intracellularly, and its presence in the culture supernatant is generally used as a measure of bacterial cell lysis (Steinmoen et al., 2002; Zafra et al., 2012). Early stationary phase cultures of the wt^lacZ^ and Δs*rfA*^lacZ^ strains were exposed to surfactin (20 μg ml^–1^ in CM medium), and CFU, eDNA concentration, and extracellular β-galactosidase activity were measured after exposure to surfactin for 0-3 h (Figure 3). Extracellular β-galactosidase activity in wt^lacZ^-conditioned medium was to approximately threefold greater than that of Δ*srfA*^lacZ^, in the absence of exogenously added surfactin. Increasing exposure to surfactin increased extracellular β-galactosidase activity of the Δ*srfA*^lacZ^ strain (Figure 3A), reaching levels similar to those in the wt^lacZ^ strain after exposure to surfactin for 3 h (Figure 3A). Increasing surfactin exposure also significantly increased eDNA concentration measured in conditioned medium of the Δ*srfA*^lacZ^ strain, but had little effect on eDNA concentration in wt^lacZ^ medium (Figure 3B). CFU showed a similar pattern, with twofold greater CFU counts in the Δ*srfA*^lacZ^ culture than in the wt^lacZ^ culture, but a subsequent decrease in Δ*srfA*^lacZ^ CFU within increasing exposure to surfactin (Figure 3C). Together, these results indicated that surfactin causes cell lysis and thus DNA release within the *B. subtilis* population.

### Surfactin-Mediated DNA Release Affects DNA Exchange

**FIGURE 4:**
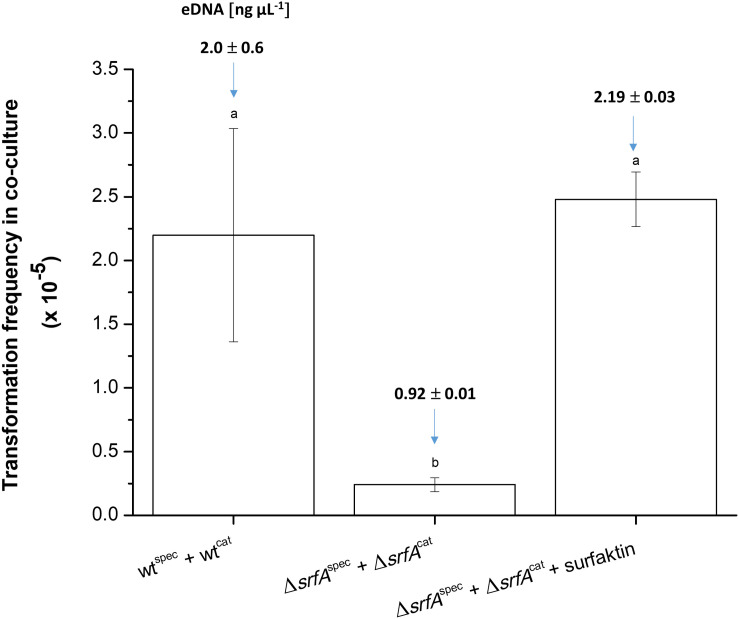
Transformation frequency in co-cultures of wt^cat^ (BM1060) + wt^spec^ (BM1058) and Δ*srfA*^cat^ (BM1062) + Δ*srfA*^spec^ (BM1063) without or with surfactin (20 μg ml^–1^) during growth in CM medium for 8 h at 37°C and 200 rpm. In each experimental variant, the eDNA concentration was determined before the transformation assay and is depicted as the number above each column. The values presented are means and standard errors (*n* = 3). Different letters above the columns indicate a statistically significant difference (*p* < 0.05) between mean values across all strains and treatments (i.e., across all columns).

The role of surfactin-mediated DNA release in DNA exchange in co-culture was then examined (Figure 4). This was determined by comparing the transformation frequency of differentially marked wild-type strains (wt + wt) and *srfA* mutants without or with added surfactin in co-cultures. When comparing transformation frequency of the Δ*srfA* mutant, it is important to note that transposon insertion in the Δ*srfA* mutant does not disrupt the *comS* gene and, therefore, should not significantly alter transformation frequency (Vollenbroich et al., 1994). This was confirmed by transforming the Δ*srfA* mutant with the wild-type strain chromosomal DNA carrying tetracycline-resistance gene (2 h after the entry into competence state), and the wild-type strain and Δ*srfA* mutant showed comparable transformation frequencies (*p* = 0.27), [(1.17 ± 0.22) × 10^–5^] and [(0.82 ± 0.16) × 10^–5^], respectively.

Next co-cultures of Δ*srfA*^cat^ (BM1062) and Δ*srfA*^spec^ (BM1063) strains were grown in the presence and absence of exogenous surfactin, and the Cat^R^Spec^R^ transformants were quantified after co-cultivation for 8 h in CM medium. The transformation frequency of the Δ*srfA* co-culture increased approximately 10-fold after surfactin addition, reaching a level similar to the wild-type co-culture (Figure 4). eDNA concentration was also measured in these co-cultures before transformation frequency was determined. eDNA concentration of the Δ*srfA* co-culture increased 2.4-fold after surfactin addition, reaching similar eDNA concentration as the wild-type co-culture (Figure 4).

## Discussion

It is well established that QS response regulators function in a pleiotropic manner by simultaneously modulating several different phenotypic traits (Schuster et al., 2003; Comella and Grossman, 2005; Antunes et al., 2007; Dandekar et al., 2012; Majerczyk et al., 2014; Wang et al., 2015). In this study, the role of the quorum-sensing regulated lipopeptide, surfactin, in the DNA release and the consequence of this action on DNA exchange between strains of *B. subtilis* were investigated. We provide evidence that DNA can be exchanged between strains without the external source of eDNA and that this HGT is dependent on eDNA released through surfactin action.

The role of eDNA in horizontal gene transfer has been previously proposed for *B. subtilis* and other naturally competent bacteria such as *Neisseria* and *Streptococcus* (Dillard and Seifert, 2001; Steinmoen et al., 2002; Zafra et al., 2012; Veening and Blokesch, 2017). It has been shown that *B. subtilis* NCIB 3610 strain releases eDNA just before entry into the stationary phase and that the Δ*srfA* mutant shows a defect in eDNA (Zafra et al., 2012), but the study did not indicate a direct role of surfactin in eDNA release. We provide this evidence that the lack of surfactin significantly impairs DNA exchange between Δ*srfA*^spec^ and Δ*srfA*^cat^ mutants, while the surfactin addition to the CM medium fully restores the transformation frequency of the Δs*rfA* mutants. Although it could be argued that the *srfA* mutant may be defective in transformation also due to ComS deficiency, we showed that this is not the case as the Δ*srfA* mutant used in this study was still transformable by externally added DNA to comparable levels as the wild-type strain. Moreover, surfactin addition to the co-culture of two *srfA* mutants restored the transformation to the wild-type levels, providing a direct evidence that this lipopeptide mediates DNA exchange, which can be abolished by DNase.

We provided here evidence that surfactin contributes to horizontal gene transfer by inducing cell lysis in a fraction of cells. It is known that surfactin producers are protected against surfactin due to resistance provided by efflux pump SwrC (YerP), which is responsible for surfactin secretion and self-resistance to surfactin (Tsuge et al., 2001). A self-tolerance against the membrane active surfactin might also be provided by the modification of the phospholipid content in the membrane of the *B. subtilis* cells (Uttlová et al., 2016). Although all cells activate *srfA* operon, over time, they show a broad heterogeneity in *srfA* expression levels (Miras and Dubnau, 2016; Dogsa et al., 2021). Moreover, although specific subpopulation undergoing lysis has not been detected, we have recently reported that *srfA* operon is already activated during the early to mid-exponential phase (Dogsa et al., 2021), between 2.5 and 3 h of growth, where strong heterogeneity in *srfA* expression is evident with a small population showing very low fluorescence of *srfA-cfp* reporter (Dogsa et al., 2021). Therefore, cells expressing *srfA* operon at very low levels might be the potential targets of surfactin-mediated lysis. We have previously observed that the membrane permeability changes during the planktonic growth of *B. subtilis*-PS-216 culture with the highest proportion of “damaged” cells (approximately 25-30%) detected around 4 h of growth (late exponential phase) (Oslizlo et al., 2014). This number dropped to only 10% in surfactin non-producers (Oslizlo et al., 2014). Although it remains to be proven whether this change in cell permeability is linked to surfactin-mediated lysis and eDNA release, this observation is consistent with the hypothesis that surfactin increases a proportion of cells with compromised membrane integrity (Deleu et al., 2013). This phenotypic change in the membrane is especially interesting because it coincides in time with the induction of the master regulator ComK and the transition to K-state (Miras and Dubnau, 2016) in 10-20% of cells (Maamar and Dubnau, 2005; Miras and Dubnau, 2016; Maier, 2020). These cells are known to be resistant to antibiotics, most probably due to growth arrest (Nester and Stocker, 1963; Engelmoer and Rozen, 2011; Yüksel et al., 2016), and therefore not targeted for surfactin lysis.

Surfactin has a broad spectrum of antimicrobial activity, and it can also lyse other *Bacilli* and non-*Bacilli* (Sharma et al., 2020), but the frequency of homologous recombination decreases sharply with the level of relatedness between the donor DNA and recipient strain’s genome (Majewski and Cohan, 1999). Therefore, we suggest that the action of surfactin may be more important for horizontal gene transfer between genetically highly related *B. subtills* strains or even within clonal populations and not between distantly related *Bacillus* strains. We have recently shown that the DNA exchange also occurs on agar surfaces at the meeting point of two swarms (Stefanic et al., 2021). However, it needs to be tested whether surfactin, which is essential for surface spreading, also contributes to eDNA release during swarming.

To conclude, we have demonstrated that surfactin contributes to horizontal gene transfer between the two almost identical populations of *B. subtilis* by inducing cell lysis in a fraction of cells. eDNA released from lysed cells then serves as the source of new genetic traits for nearby competent cells or for DNA repair, if exchange occurs within clonal population. Therefore, surfactin is linked to horizontal gene transfer at the level of its operon, which embeds ComS and, as a pore-forming lipopeptide, that contributes to eDNA pool.

## Data Availability Statement

The original contributions presented in the study are included in the article/supplementary material, further inquiries can be directed to the corresponding author/s.

## Author Contributions

TD, AD, and IM-M conceived and designed the experiments. TD, AD, MS, and PS performed the experiments. TD, AD, MS, PS, and ID analyzed the data. TD, AD, PS, and IM-M wrote the manuscript. All authors contributed to the final version of the manuscript.

## Conflict of Interest

The authors declare that the research was conducted in the absence of any commercial or financial relationships that could be construed as a potential conflict of interest.
